# The student perspective on teaching and assessment during initial COVID-19 related closures at Irish universities: implications for the future

**DOI:** 10.1093/teamat/hrab017

**Published:** 2021-10-04

**Authors:** Diarmaid Hyland, Ann O’Shea

**Affiliations:** Department of Mathematics and Statistics, Maynooth University, Maynooth, Co. Kildare, Ireland

## Abstract

The aim of this research is to investigate the impact of the COVID-19 closures from the perspective of students studying mathematics at university level in Ireland. A survey was designed and administered to students who were enrolled in at least one mathematics module in an Irish university at the time of the closures. The survey comprised three sections: teaching and learning, assessment and personal experience, with a focus on how the changes in teaching and assessment were viewed by students. A total of 263 students from six universities responded to the survey. The corresponding data are described, as are various trends that were identified through open-response questions. The data offer a window into the student experience during the tertiary level closures and show the effect that the sudden shifts (e.g., in delivery and support) had on learning, assessment and student well-being. The survey responses show that most students dealt with the rapid changes in a resilient and mature manner, particularly when confronted with adversity. Numerous insights can be gleaned from the students’ perspectives that have the potential to improve the teaching and learning of mathematics at tertiary level in Ireland in the future. Many of the findings could also apply to teaching and learning in other subjects and internationally.

## 1 Introduction

The challenges associated with COVID-19 are unquestionably the largest to face the mathematics education community in recent times. In Ireland, the government announced the closure of schools, including universities and institutes of technology on 13 March 2020. Tertiary level institutions remained closed for the 2019–2020 academic year, with all teaching and assessment done remotely. The abrupt nature of the measures introduced by governments to combat the spread of the disease, combined with continued measures to minimize its spread, has placed significant logistical constraints on educators, support staff and students.

Crawford *et al.* (2020) explored the first wave of responses of universities to the COVID-19 pandemic in perhaps the most wide-reaching study to date. They looked at 172 sources from 20 countries (including Ireland) to arrive at their findings. They found that most countries moved to fully online instruction during the spring semester in 2020 (March to May). Therefore, the situation in Irish universities has much in common with that of the rest of the world.

Prior to the pandemic, there had been an increase in the use of e-learning in universities, and over the past 10 years, many researchers have studied online instruction in undergraduate mathematics courses. Trenholm & Peschke (2020) outline six differences in execution between fully online and face-to-face instruction in mathematics communities of practice at tertiary level. Among the differences they found is the notion that the content being learned is the same as before, but significant changes in communication, interaction and assessment must occur when transitioning from face-to-face to fully online learning (Trenholm & Peschke, 2020). In particular, they note that fully online learning puts a greater responsibility on students to read or view resources themselves rather than be guided through material in a teacher-led setting. Trenholm & Peschke (2020) also note that mathematics notation is often a barrier to discussion since it is difficult for students to typeset symbols in emails or on forums. Trenholm & Peschke (2020) point out that the timing of interactions in fully online courses can be quite different to that in face-to-face modules as students may have to wait a considerable time to get a response to a question in an email; however, this also allows opportunities for students to reflect. A review of research on online learning in mathematics was carried out by Engelbrecht *et al.* (2020). In this work, the authors investigate how the mathematics classroom has evolved with the growth of the internet and interactive devices. The authors outline how rapidly evolving technology has spawned micro-generations of students who have grown up in an increasingly technology-immersed world. Dineva *et al.* (2019) explain that despite differences between the micro-generations, they share strong core characteristics: students today have digital profiles on multiple social networks where they learn how to interact (Jukes *et al.*, 2010); they are good multi-taskers who want information to be delivered in dynamic ways (Dineva *et al.*, 2019), often having procured it themselves as opposed to it being handed to them (Morin, 2016); and they collaborate well, often utilizing technology in the process (Engelbrecht *et al.*, 2020). Morin (2016) explains these learners have their own concerns, new motivations and new challenges with respect to education; the solutions to which Dineva *et al.* (2019) explain may lie in the increased capacity for interactivity and learning afforded by technology entering the classroom. We note, however, that research on massive open online courses, blended learning, flipped classrooms or other technologically progressive methods in use prior to COVID-19 might not be as applicable to current teaching as one might expect. Despite the fact that this work is undoubtedly instructive for practitioners who seek to move their teaching in this direction in the future, we consider it to be noticeably different to post-COVID-19 instruction. Engelbrecht *et al.* (2020) noted that many instructors were forced to move their teaching online very suddenly in Spring 2020 and this led the to a ‘domestication of new media’—that is, the traditional versions of courses were used as models for the online versions without taking full advantage of the new opportunities afforded by the technology.

Perhaps a more accurate description of what unfolded in March 2020 is emergency remote teaching, which is described by Hodges *et al.* (2020) as follows:

a temporary shift of instructional delivery to an alternate delivery mode due to crisis circumstances. It involves the use of fully remote teaching solutions for instruction or education that would otherwise be delivered face-to-face or as blended or hybrid courses and that will return to that format once the crisis or emergency has abated. (Hodges *et al.*, 2020, p. 6)

The student perspective of emergency remote teaching is arguably under reported internationally to date. Unger & Meiran (2020) investigated student perceptions and attitudes towards higher education distance learning during the pandemic in the USA ‘at a small undergraduate university’. They found significant anxiety and reduced performance among their students brought about by the transition to online learning. Similarly, in an interview study of students in an Indian university, Chattaraj & Vijayaraghavan (2021, p. 9) reported student accounts of ‘strangeness and lack of familiarity with the experiences of learning and learning space in Emergency Remote Learning’. Adnan & Anwar (2020) surveyed students concerning technology and infrastructure in Pakistan and reported similar dissatisfaction among students, with 78% of their respondents agreeing that face-to-face contact with the instructor is necessary for learning.

In this paper, we will describe the design and subsequent results of a survey (Appendix A) given to students who were enrolled in mathematics modules in Irish universities during Semester 2 (March to May) of the 2019–2020 academic year. The perspective of mathematics learners internationally to the COVID-19 closures is as yet under-reported, though a similar survey to ours was carried out by members of the School of Mathematics and Statistics at University College Dublin. Their results were published as an internal report (Meehan & Howard, 2020) and disseminated as a public lecture (10 May 2020). The study focused on students taking a mathematics module at University College Dublin at the time of the closures. The researchers reported on the positives (*commuting*, *self-pacing*) and negatives (*internet connection*, *lack of peer interaction*) that students associated with distance learning. They also reported that students perceived the quality of traditional lectures to remain relatively constant, whereas they perceived the quality of lectures that were more interactive prior to the closure to lessen following the move online. This is an issue with pedagogies for distance learning which was identified by the International Association of Universities Global Survey (Marinoni *et al.*, 2020) which is not surprising given the difficulty of maintaining student interaction in the online setting. Apart from the University College Dublin study, research in this area in Ireland has focussed on the perspective of the lecturers (Ní Fhloinn & Fitzmaurice, 2021) and support staff (Irish Mathematics Learning Support Network, 2021) in mathematics and on second-level science teachers (Chadwick *et al.*, 2021) but has yet (insofar as we can ascertain) to investigate the student experience and their perceptions of higher education during the pandemic. The student perspective was captured in an article by two nursing students in Ireland, however, who report reduced motivation and a ‘feeling of disconnectedness and isolation’ brought about by a reduction in engagement between students and lecturers (Hill & Fitzgerald, 2020, p. 3).

We believe that students are the most important stakeholders in mathematics education and that their experiences and opinions will offer valuable insights into how the closures were handled and how best to proceed with mathematics education in an ever-changing landscape. The aim of the study is to investigate the impact of the initial COVID-19 tertiary level closures has had on students studying mathematics in Irish universities. The research questions are as follows:

1. What were university mathematics students’ experiences of teaching, studying and assessment during the initial COVID-19 closures?
2. What are mathematics students’ views on the provision of future teaching and assessment?

Answering these research questions will give us a clear picture of the practices that were adopted to continue the teaching and learning of mathematics following the tertiary level closures in March 2020 and how they impacted on student learning. Insights on assessment techniques and future preferences for teaching and learning may be useful to practitioners as they adapt their approaches in subsequent modules. In addition, data relating to the students’ personal experiences can inform student support services both in mathematics and in general. In the following sections, we outline the methods of data collection and analysis, present the results of the study and discuss the findings in the ever-evolving context of tertiary level education during the COVID-19 pandemic.

## 2 Methodology

In this section, we describe the collection and analysis of the data in this study.

### 2.1. Data collection

We designed a survey (Appendix A) to inform the research questions. The survey comprised 16 questions, divided into three sections: teaching and learning, assessment and personal experience. It consisted of a combination of closed questions (Likert scale items or ‘tick box’ responses) and some open response questions. The survey was advertised primarily through email. It was open from 9 July to 9 August 2020 and as such only pertained to the students’ experience from 13 March until the end of semester at their institution. This included any learning and assessment during the second semester of the 2019–2020 academic year but does not cover subsequent learning and assessment associated with the 2020–2021 academic year, although this has also occurred under restrictions. The authors forwarded the survey to tertiary level mathematics lecturers in Ireland through various mailing lists and many of the lecturers forwarded the survey to their students.

### 2.2. Participants

**Fig. 1.**
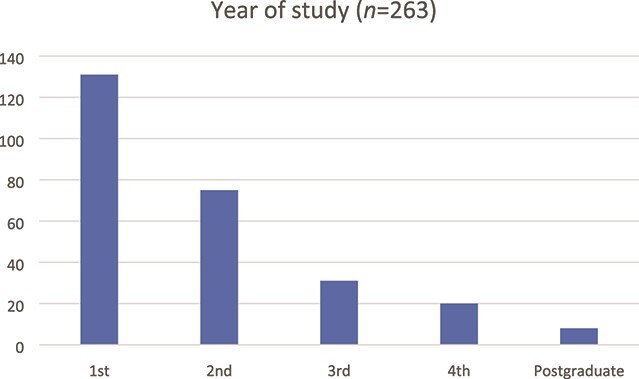
Year of study of participants.

In total, 263 students from six universities responded to the survey. A plain language statement preceded the survey and informed consent was received from all 263 students reported in this study. The survey was shared with students from all tertiary level institutions, but a significant majority of respondents (93% of 282) were university students. This is not an optimal representation of the balance between university students and institute of technology students in Ireland, though it is understandable because of the restrictions in place in institutes of technology during summer months that affected staff members’ ability to forward the survey to their students. Therefore, we decided to omit the responses from the small number of institute of technology students. The year of study for respondents is shown below (Fig. 1).

The majority of respondents were enrolled in the authors’ university (62%), with the remainder spread across five of the seven other Irish universities. The most frequent degree programmes represented in our sample were specialist mathematics courses (21%), teacher education (20%) and general science (19%). Students studying a single science subject (e.g., physics, chemistry or biology) made up 13% of the respondents, while 11% of the group were studying mathematics as part of a BA programme and 7% were enrolled in computing degrees. The even spread reported across degree programmes is desirable because it implies that the data pertain to a wide range of modules, making it a more accurate reflection of the situation at large. Similarly, respondents pursuing a degree in mathematics will have been enrolled in multiple mathematics modules during closures. This means that their answers to many of the questions (Q1, 2, 5, 6–11, 13–16) are informed by multiple experiences.

### 2.3. Data analysis

A combination of qualitative and quantitative data was collected from survey responses. The quantitative data gathered in response to the closed response questions were tallied and are presented in tables in the Results section. We analysed the responses to the open questions by following the general inductive approach to qualitative data analysis outlined by Thomas (2006). This approach allows ‘research findings to emerge from the frequent, dominant or significant themes inherent in raw data, without the restraints imposed by structured methodologies’ (2006, p. 283). The process, which uses coding to develop categories to condense the data, allows links between the research questions and the findings to be established (Thomas, 2006). Procedures to assess the trustworthiness of the category system (independent coding, coding consistency check and stakeholder checks), which Thomas (2006) describes, were also practiced during our data analysis.

Thomas’ (2006) general inductive approach was used in the analysis of the responses to 11 open questions on the survey. The analysis began with the preparation of the raw data files which were read and interpreted multiple times by the authors independently. Once this was done, each author identified the themes in the data and defined categories on a question-by-question basis. The authors’ categories were combined to form an exhaustive list. We refined this list by removing duplicates and grouping very similar categories together. Each of the categories were reduced to a word or short phrase (label of category) encapsulating their meaning. The student responses were then categorized by each researcher independently before a meeting took place to discuss outliers and other discrepancies.

Multiple meetings took place, always yielding a refined categorization and improved inter-rater agreement until complete agreement was reached. In the initial round of coding, there was 90% agreement between the researchers. In the final iteration (where all 1712 student responses were coded), 157 categories were used across 11 questions. Within the analysis, many responses were coded into more than one category while others remained uncoded which is in keeping with Thomas’ (2006) approach. Individual responses can relate to more than one category due to their detail or comprehensiveness. Similarly, responses can remain uncoded if they are too vague or unrelated to the question.

## 3 Results

In this section, the results of the study are presented; 263 students from six universities responded to the survey in full. The research questions focused on the students’ experiences of teaching, studying and assessment during the closures in Spring 2020. The views of students on how best to teach and assess mathematics in subsequent modules were also sought. We begin by looking at the data on students’ access to technology and suitable workspaces which might impact their ability to work remotely.

### 3.1. Capacity to learn remotely

Five closed questions were designed to learn about the equipment, infrastructure and facilities students had access to while learning from home. The results (Table 1) show that the vast majority of students had access to the appropriate equipment to enable them to study. This was not true of all students; however, there are economic and/or geographical factors that restrict access to equipment (table, computer, scanner), infrastructure (fast, reliable broadband) and facilities (quiet space). It is notable that more than one third of students did not have reliable broadband and a similar number did not have access to a printer/scanner. In addition, many students use apps on their phone to scan their work which we believe impacted the responses to Q12 (iv) in Table 1.

**Table 1:** Students’ access to equipment, infrastructure and facilities for distance learning (Q12)

Q12 g–k: access to equipment, infrastructure and facilities		Yes	No	Prefer not to say
g	Did you have access to a quiet place to study?	200 (76%)	62 (23.6%)	1 (0.4%)
h	Did you have access to a table/desk?	249 (94.7%)	13 (4.9%)	1 (0.4%)
i	Did you have access to a PC or laptop?	256 (97.3%)	6 (2.3%)	1 (0.4%)
j	Did you have access to a printer/scanner?	186 (70.7%)	75 (28.5%)	2 (0.8%)
k	Did you have access to fast and reliable broadband?	166 (63.1%)	90 (36.5%)	1 (0.4%)

### 3.2. Students’ experience of teaching, studying and assessment during the Spring 2020 closures

Ten questions on the survey informed our first research question. They included open and closed questions that probed the nature of teaching, studying and assessment during the Spring 2020 closures, including the prevalence of different supports and resources.

#### 3.2.1. Lectures and resources

We begin by describing how lectures and tutorials were delivered after the transition to distance learning; we detail the way students had to adapt their learning, before discussing the impact of the closures on mathematics support services.

The opening question of the survey asked students about the methods used to deliver their lectures following the restrictions on attending campus. Nine options (those in Table 2) were given to students as a list where they were asked to select every method utilized by their lecturers. The results show the wide range of approaches adopted by lecturers. The use of online shared spaces or virtual learning environments (VLEs) (e.g., Moodle, Blackboard) to upload a variety of resources was the most popular method of delivery. Live or recorded presentations and screencasts were commonly used, and some students mentioned that they were directed to external resources (e.g., YouTube, Khan Academy). We note that we do not have information on the extent to which the different teaching methods were used in modules.

**Table 2:** Teaching methods used during the COVID-19 closures (Q1)

Q1: method employed by lecturer	Tally: number of responses (percentage of cohort)
Recorded lectures uploaded to a shared space	226 (85.9%)
Written notes uploaded to a shared space	211 (80.2%)
Worked examples uploaded to a shared space	160 (60.8%)
Practice quizzes	140 (53.2%)
Live lectures hosted online	123 (46.8%)
Assignments/past exam solutions uploaded to a shared space	121 (46%)
PowerPoint (or similar) uploaded to a shared space	116 (44.1%)
Short videos with specific focus uploaded to a shared space	96 (36.5%)
Directed to various online resources that already existed	49 (18.6%)
None of the above	1 (0.4%)

The vast majority of students reported that their lecturers provided notes through their university’s VLE and also recorded the lectures or created short videos to complement the notes; it may be that lecturers adapted their pre-existing teaching resources during the pivot to fully online delivery. Most of the resources created were delivered in an asynchronous manner with just under half of the respondents saying that they had live online lectures. Another striking feature of the responses to this question is the small number of students (18.6%) who said that they were directed to existing online material. This means that lecturers created a huge amount of online content in a very short time period. Many of the students reported that their lecturers (in addition to providing content through lectures or notes) also created practice quizzes and worked examples and made solutions to assignments and past examination questions available.

However, even though these resources were made available to students, the responses to Q3 of our survey (Table 3) show that students struggled with the move from face-to-face to remote learning. About 59% of them said that the closures had a negative impact on their capacity to learn mathematics, with only 21.3% saying that the impact was positive. Students reported negative effects on their ability to learn through lectures (59.4%), tutorials (65.8%) and mathematics support services (54.7%). They also found it more difficult to communicate with their peers (57.3%) or to engage in peer study (65.5%). There are signs though that the students found ways to adapt to the situation, for example 58.2% of them either said that the COVID-19 closures had no impact on their ability to complete assessments or that it had a positive impact.

**Table 3:** How the closures impacted students (Q3)

Q3: how have the COVID-19 third-level^2^ closures impacted your capacity to						
	*Very negatively*	*Somewhat negatively*	*No impact*	*Somewhat positively*	*Very positively*	*Not applicable*
Learn mathematics generally	12.5% (*n* = 33)	46.4% (*n =* 122)	19.8% (*n =* 52)	15.2% (*n =* 40)	6.1% (*n =* 16)	0
Learn through lectures	20.2% (*n =* 53)	39.2% (*n =* 103)	19.4% (*n =* 51)	14.1% (*n =* 37)	6.5% (*n =* 17)	0.8% (*n =* 2)
Learn through tutorials	36.3% (*n =* 102)	29.5% (*n =* 83)	20.6% (*n =* 58)	6.4% (*n =* 18)	3.9% (*n =* 11)	3.2% (*n =* 9)
Learn through mathematics support services	30.4% (*n =* 80)	24.3% (*n =* 64)	30.4% (*n =* 80)	6.1% (*n =* 16)	4.6% (*n =* 12)	4.2% (*n =* 11)
Independent study	9.9% (*n =* 26)	16% (*n =* 42)	23.6% (*n =* 62)	27.4% (*n =* 72)	22.4% (*n =* 59)	0.8% (*n =* 2)
Peer study	37.9% (*n =* 99)	27.6% (*n =* 72)	20.7% (*n =* 54)	8.4% (*n =* 22)	3.4% (*n =* 9)	1.9% (*n =* 5)
Communicate with your lecturer	12.2% (*n =* 32)	27.8% (*n =* 73)	39.2% (*n =* 103)	17.1% (*n =* 45)	3.8% (*n =* 10)	0
Communicate with your peers	22.9% (*n =* 60)	34.4% (*n =* 90)	23.3% (*n =* 61)	11.5% (*n =* 30)	7.6% (*n =* 20)	0.4% (*n =* 1)
Communicate with support services	18.6% (*n =* 49)	24.3% (*n =* 64)	44.5% (*n =* 117)	5.7% (*n =* 15)	2.3% (*n =* 6)	4.6% (*n =* 12)
Complete assignments and other forms of assessment	12.5% (*n =* 33)	28.9% (*n =* 76)	30.4% (*n =* 80)	16% (*n =* 42)	11.8% (*n =* 31)	0.4% (*n =* 1)

**Table 4:** The resources that students used to study (Q4)

Q4: how have the COVID-19 third-level closures impacted how often you use the following resources to support your learning:						
	*Very negatively*	*Somewhat negatively*	*No impact*	*Somewhat positively*	*Very positively*	*Not applicable*
Lectures	16.3% (*n =* 42)	30.6% (*n =* 79)	31% (*n =* 80)	15.1% (*n = 39*)	6.2% (*n =* 16)	0.8% (*n =* 2)
Lecture notes provided by lecturer	5.3% (*n =* 14)	17.9% (*n =* 47)	28.2% (*n =* 74)	34% (*n =* 89)	13% (*n =* 34)	1.8% (*n =* 4)
Other resources provided by lecturer	6.5% (*n =* 17)	14.8% (*n =* 39)	30.4% (*n =* 80)	35% (*n =* 92)	12.2% (*n =* 32)	1.1% (*n =* 3)
Mathematics support services	31.3% (*n =* 82)	24.4% (*n =* 64)	28.2% (*n =* 74)	8.4% (*n =* 22)	2.7% (*n =* 7)	5% (*n =* 13)
Prior exam questions	10.3% (*n =* 27)	14.4% (*n =* 38)	49.4% (*n =* 130)	17.1% (*n =* 45)	6.8% (*n =* 18)	1.9% (*n =* 5)
Practice quizzes	6.5% (*n =* 17)	11.1% (*n =* 29)	41.8% (*n =* 109)	25.7% (*n =* 67)	11.1% (*n =* 29)	3.8% (*n =* 10)
Online resources (external)	4.6% (*n =* 12)	6.5% (*n =* 17)	47.9% (*n =* 126)	19.4% (*n =* 51)	14.8% (*n =* 39)	6.8% (*n =* 18)
Books	19.1% (*n =* 50)	19.1% (*n =* 50)	43.1% (*n =* 113)	11.8% (*n =* 31)	2.3% (*n =* 6)	4.6% (*n =* 12)

We used chi-square tests to examine the relationships between the teaching methods of lecturers and the students’ reports of how the closures impacted on their learning (Q3). We found that students who had access to solutions to assignments and exam papers were more likely to be positive about the impact of the closures on their capacity to learn mathematics generally (*p* = 0.004) than those who did not have access to solutions. Similarly, we found that students who had access to recordings of lectures were more likely to feel that the closures had a positive impact on their ability to learn through lectures (*p* = 0.005) than students who did not. However, we found that there were no significant differences in the distribution of responses to Q3 between students who had live lectures and those who did not.

We asked the students how the COVID-19 closures impacted the frequency with which they used resources (Table 4); the responses mirrored those of Q3. Some students reported less frequent use of mathematics support services (55.7%) and lectures (46.9%); both of which are understandable given the face-to-face nature of these teaching methods. However, they also reported increased use of notes and other resources provided by the lecturer (both 47%). It is interesting to note that for each of the categories mentioned in Q4, quite a few students reported that the closures had no impact on how often they made use of the resources.

#### 3.2.2. Tutorials

A large proportion of respondents (76.8%, *n* = 202) had tutorials during Spring 2020, though much of these were significantly hampered by the restrictions. Respondents were asked to detail the support that was available to them in a subsequent open-ended question (Q2), to which 178 students (68%) responded. Twenty students had their tutorials replaced by online discussion forums and a further 20 were given a point of contact for forwarding questions. The students did not always find forwarding questions effective, for example one student said the following:

Student 168: You could email questions but hard to understand a reply.

Over two-thirds of these students (71%) had access to live Zoom or Microsoft Teams sessions with a tutor. In general, students seemed to prefer the live tutorials to online forums:

Student 161: Originally no tutorials were offered but instead a open forum where students could ask tutors questions and everyone else would see these interactions. Then only after a request, live tutorials, on Microsoft Teams, were arranged, which were optional but very beneficial. I would’ve been lost without them!

However, even when online tutorials were available, students felt that they did not work in the same way as regular tutorials:

Student 62: There was question and answer sessions available, but they were very different to our usual tutorials. In person tutorials in (name of university) are in a small class where the tutor explains the homework, and goes through questions with you, encourages you and gives tips. On the online sessions, in my experience, you had to come prepared with a question about the homework, which assumes you already sort of know whats going on. If nobody had questions we would just wait around until someone asked one.

Other supports (in lieu of live online tutorials) were available to some students: 31 students (16%) reported that they had access to resources uploaded to a VLE, consisting of solutions to problem sets (*n* = 10), recorded presentations (*n* = 17) and notes (*n* = 4). Just four students mentioned receiving feedback on work they submitted.

Though the proportion of students having access to tutorials seems relatively large (at almost 77%) considering how abruptly in-person delivery halted, we would caution readers to consider the nature of the provisions. Much of what was offered to students was asynchronous in the form of solutions to problems sets, short video clips, answers to questions on a forum, etc. What the students seemed to miss were the personal interactions which are possible in small-group tutorials. Along similar lines, even the students for whom tutorials were available deemed the move online as having a negative impact (62.4% of respondents) on their ability to learn through tutorials.

#### 3.2.3. Mathematics support services

Despite pre-COVID increases in provision of mathematics support online (Mac an Bhaird *et al.*, 2020), support services nationally were adversely affected by the university closures with institutions experiencing varying levels of success during COVID-19 (Irish Mathematics Learning Support Network, 2021). Such success relied on support services quickly establishing and maintaining an online presence, but they were still unable to provide the same experience as in-person support. Support for this is found in this study, for instance one respondent said the following:

Student 92: The 1 on 1 support from tutorials and maths support centre is very hard to recreate online. Lots of work can be self directed however an hour or 2 of in person teaching can really help.

This sentiment is supported by the results to Q3 d, which related to how the Spring 2020 closures impacted students’ ability to learn through mathematics support centres. A total of 56% of students responded negatively to this question, with ‘very negative’ being the most common response. Many students in this survey mentioned the difficulty of maintaining the atmosphere of mathematics support once it moved online.

There appears to be a compounding impact of the closure of support spaces. In recent years, many students have been using these spaces (often centrally located on campus) for peer study and not strictly for direct support:

Student 179: No access to library (in particular [name of university]’s maths learning center) and comparing solutions to other classmates really hindered my exam preparation.

In this respect, the positive aspects of mathematics support incurred an additional, ‘hidden’ impact on a significant number of students who benefit from more than tutor support. With respect to peer study, the students returned the most negative impact recorded on Q3 or Q4 (Tables 3 and 4), with 38% of students reporting that the closures impacted their ability to study with peers ‘very negatively’.

#### 3.2.4. Assessment

The survey questions that concerned assessment are Q6–10 inclusive. The questions included open and closed parts that investigated the types of assessment practices that students had to engage with, in addition to how they prepared for their assessments.

**Fig. 2.**
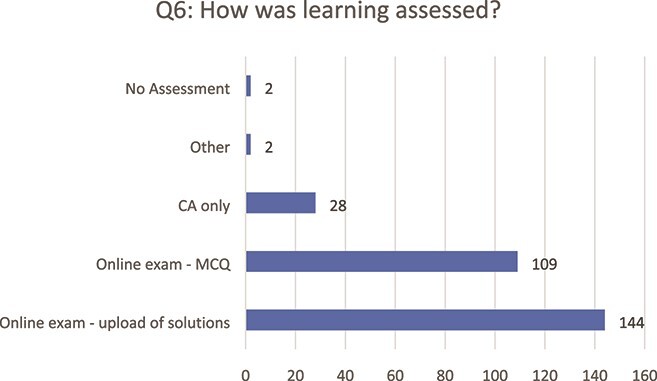
Methods used to assess students’ learning (Q6).

We begin by investigating what methods of assessment lecturers used following the transition to online learning (Fig. 2). The majority of learners (90%) were assessed using terminal online exams, either with multiple choice questions (including open response questions) or with upload of solutions. This number includes modules where terminal exams were used in conjunction with continuous assessment. The use of continuous assessment only was far less frequent, with only 28 students (10%) reporting being assessed in this manner. Two students reported no terminal assessment and a further two were not sufficiently clear for their responses to be labelled appropriately.

A significant majority of the students (80%) considered the assessment to be fair (albeit unconventional), given the circumstances (Q7). However, areas for improvement with respect to assessment were also identified: only 43% of students said that they received feedback on any submitted work, and almost a quarter of students (24%) had technical difficulties during their final assessment.

The issues cited by students in relation to technical difficulties in final examinations were predominantly related to internet connection (*n* = 18), software (*n* = 10) and computer/scanner issues (*n* = 8). In addition, seven students highlighted shortcomings in the planning and execution of online exams, with the following excerpt being typical of this:

Student 27: Nowhere near enough time given to complete the online exam and also no extra time was given to upload solutions like in other modules.

More students mentioned exams that appeared in an illegible format for students when the exam went live, while another student reported a significant amount of time was spent identifying students during a Zoom invigilation because they were not pre-instructed to use a specific username.

Given the changes in teaching and assessment, we were interested to see if students adapted the way they prepared for their examinations. While typical modes were still used to prepare, there was evidence of novel approaches that reflect the unique circumstances students faced. With respect to pre-pandemic approaches to studying, students reported practicing *past exam questions* (*n* = 92; 38.3%), completing tutorial *questions* (*n* = 79; 32.9%), using *lecture notes* (*n* = 76; 31.7%), using other *external resources* (e.g., YouTube, Khan Academy) (*n* = 26; 10.8%) and books (*n* = 7; 2.9%). In fact, 25 students (10.4%) stated explicitly that they prepared for these examinations in the same way that they approached examinations in the past. The other approaches reported by students have clear connections to the changes in the delivery and assessment of learning. Forty students (16.7%) rewatched the recorded lectures as part of their preparation, 15 students (6.3%) devoted time to preparing for the open book nature of their exam and three students (1.3%) practiced uploading files ahead of their assessment.

**Table 5:** Student data on anxiety, isolation, and motivation (Q12)

Q12 d–f: extent to which students experienced anxiety, isolation and lack of motivation while learning remotely							
		*Strongly agree*	*Somewhat agree*	*Neutral*	*Somewhat disagree*	*Strongly disagree*	*Prefer not to say*
d	It was easier to motivate myself to learn during the lockdown than before	8% (*n =* 21)	8.7% (*n =* 23)	14.8% (*n =* 39)	25.1% (*n = 66*)	39.2% (*n =* 103)	4.2% (*n =* 11)
e	I felt more anxious about my learning during the lockdown than before	32.7% (*n =* 86)	29.7% (*n =* 78)	15.6% (*n =* 41)	11.8% (*n =* 31)	9.1% (*n =* 24)	1.1% (*n =* 3)
f	I felt isolated from my lecturer/class group	29.7% (*n =* 78)	26.6% (*n =* 70)	22.8% (*n =* 60)	10.3% (*n =* 27)	10.3% (*n =* 27)	0.4% (*n =* 1)

### 3.3. Students’ experience of the transition to distance learning

Included in the survey were several open response questions that sought to investigate the students’ personal experiences surrounding the transition to distance learning (Q12). There were 11 parts to this question included in the analysis; we have already seen the data from the five parts in Table 1. We discuss the responses to three of the sub-questions here (and another three in Table 9), beginning with expressions of motivation, anxiety and isolation.

Students reported feeling increased anxiety and isolation during the Covid-19 closures. This is to be expected with such a sudden switch from in person to distance learning but is none the less worrying. Of the 263 students who responded to the survey, 164 (62%) reported increased anxiety, 148 (56%) felt isolated from their lecturers and peers and 169 (64%) students believed it was more difficult to motivate themselves during lockdown. Strategies for addressing isolation were sought; many responses suggested ways of increasing contact among students while acknowledging the difficulty of resolving such an issue in the current environment. The increased isolation is also seen in responses to parts of Q3 (Table 3), where students reported negative effects on communication with lecturers, peers and support services and a very negative impact on their capacity to peer study which recurs throughout the data. We did not see statistically significant relationships between the teaching methods experienced by students and their levels of anxiety. However, we did see that students who had access to solutions to worked examples (chi-square test, *p* = 0.048) and practice quizzes (*p* = 0.003) were more likely to agree that it was easier to motivate themselves than students who did not have access to these resources. We also saw that students who reported having solutions to worked examples were less likely to feel isolated (*p* = 0.024). Surprisingly, we did not see relationships between whether students had live lectures or not and the motivation, anxiety and isolation variables.

We asked three open response questions about the challenging (Q14) and positive (Q15) aspects of teaching, learning and assessment during the tertiary level closures and asked for further comment (Q16) which are presented below (in Tables 6–8, respectively). Categories with five tallies or fewer were omitted.

We coded the challenges students reported into five overarching categories: *delivery of teaching* (*n* = 90), *interaction and communication* (*n* = 197), *assessment* (*n* = 20), *motivation* (*n* = 75) and *learning environment* (*n* = 21). The codes that contributed to our categories are listed in Table 6. The category *interaction and communication* was the most prevalent showing that this was the main challenge confronting these students; furthermore, the codes that contributed to the *delivery of teaching* category also dealt with loss of interaction with instructors.

Students reported difficulties with motivating themselves and with self-pacing. While student motivation can be impacted by many factors, it is understandable that the toll of the pandemic was felt by students:

Student 58: very hard to motivate yourself to learn during a global pandemic. Maths seems frivolous.

Surprisingly, only 20 comments were made about assessment. Within these, the online *format of the assessments* was mentioned by 13 students and inputting *mathematical notation online* (*n* = 3), *submitting assessments* (*n* = 2), *the open book nature of the assessment* (*n* = 1) and simply ‘assessment’ (*n* = 1) were also mentioned. A common thread to many of these comments is the air of uncertainty that surrounded much of the process, captured by the following comment:

Student 59: The worst part easily was the not knowing of how things were going to come together at the end. It’s understandable that the situation was ever changing but not being advised as to how we would be taking our exams or what we had to study up until the month of the exams was just as stressful.

Finally, challenges with the learning environment were mentioned by students, comprising of having access to an appropriate *study space* (*n* = 13) and issues with *technology or internet* (*n* = 8).

The common theme uniting many of the most frequently occurring labels is the social aspect of learning. This disconnect led to issues with *motivation* and *isolation* which, when added together, total 73% of the responses to this question.

**Table 6:** Challenging aspects of changes mentioned by students (Q14)

Q14: in your opinion, what have been the challenging aspects of learning, teaching and assessment in mathematics during the COVID-19 third-level closures?		
Category	Label	Tally
Delivery of teaching	Loss of in-person delivery	28
	Access to support (e.g., MSC/MLC)	54
	Access to resources (e.g., books)	8
Interaction and communication	Less communication with lecturer	16
	Peer interaction	46
	Asking questions in real time	30
Assessment	Assessment format	13
Motivation	Motivation	52
	Self-pacing	17
Learning environment	Home space	13
	Technology and internet	8
Other		15

There were also several positive aspects to teaching and learning that students reported, outlined in Table 7.

**Table 7:** Positive aspects of changes mentioned by students (Q15)

Q15: in your opinion, what have been the positive aspects of learning, teaching and assessment in mathematics during the COVID-19 third-level closures?		
Category	Label	Tally
Learning	Self-pacing	44
	Individual study skills	26
Time	No Commute	35
	No set timetable	38
Resources	More internal resources (e.g., module specific notes, solutions to problems provided by lecturers and/or tutors)	55
	Found external resources (e.g., third-party websites with useful resources)	7
Assessment	Open book	8
	Online assessment	6
Other		14

An obvious benefit to learning from home was the amount of time and money students saved on commuting to and from campus (*n* = 35) and on rent. The lack of a set timetable (*n* = 38) for many students allowed them to work at their own pace (*n* = 44) and at a time that best suited their situation. Similarly, students accessed a more varied range of resources (both internally (*n* = 55) and externally (*n* = 7)).

There were 77 responses to the final question of the survey, which called for any additional feedback students wanted to submit (Table 8). The majority of these are related to student’s personal experiences (*n* = 33) and teaching and learning (*n* = 34), with a further eight comments specifically related to assessment. Students’ personal experiences varied widely, as is to be expected given the unprecedented nature of the pandemic, with the following excerpts typical of a positive and negative experience, respectively:

Student 80: Overall the [University name] maths department coped well with the situation. I felt everyone did their best and were very accommodating.

Student 18: I understand why it was done the way it was done, but it was extremely stressful for me. I was so stressed and I had so much going for me having my own study room, laptop and scanner, so I can’t imagine how stressed less fortunate people were.

**Table 8:** All remaining comments mentioned by students (Q16)

Q16: please share any remaining comments and opinions you may have relating to the COVID-19 third-level closures and their impact on your learning of mathematics.		
Category	Label	Tally
Teaching and learning	Mathematics is a social subject/needs to be delivered in person	11
	More/All resources should be uploaded even when in-person delivery returns	7
	Clarify standards across departments/institution	6
Personal experience	Negative experience	8
	Resilient and pragmatic viewpoint	8
	Positive experience	6
Assessment	Assessments need to be improved and standardized	6

### 3.4. Students’ views on the provision of future teaching and assessment

Returning to Q12 on students’ personal experience, we asked students to indicate how comfortable they would be with attending lectures, tutorials and support centres on campus; the results are given in Table 9. About two-thirds of students would have been comfortable attending classes on campus as long as appropriate precautions were taken.

**Table 9:** Students’ comfort with returning to campus for delivery of teaching in future (Q12)

Q12 a–c: Students’ feeling towards a return to campus (where the appropriate precautions are taken)							
		*Strongly agree*	*Somewhat agree*	*Neutral*	*Somewhat disagree*	*Strongly disagree*	*Prefer not to say*
a	I feel comfortable attending lectures on campus next year	42.6% (*n =* 112)	28.5% (*n =* 75)	5.3% (*n =* 14)	16% (*n = 42*)	6.8% (*n =* 18)	0.8% (*n =* 2)
b	I feel comfortable attending tutorials on campus next year	48.3% (*n =* 127)	25.1% (*n =* 66)	9.1% (*n =* 24)	12.9% (*n =* 34)	3.8% (*n =* 10)	0.8% (*n =* 2)
c	I feel comfortable attending mathematics support services on campus next year	43.3% (*n =* 114)	24% (*n =* 63)	14.8% (*n =* 39)	12.5% (*n =* 33)	4.2% (*n =* 11)	1.1% (*n =* 3)

We conclude with the results to an open-ended question (Q5) that asked students about their preferences for teaching methods for the year ahead; 238 students responded to this question^1^. Of these, 71 students (30%) said that they wanted all teaching to take place on campus, 112 (47%) said that they would like teaching to take place online and 20 (8.5%) favoured a blended approach. Two (0.8%) students said that they would like teaching to be either 100% online or 100% in person. The remaining respondents did not clearly indicate whether they wanted instruction to be online or in person; of these, 14 (5.8%) students asked for worked examples or exam solutions to be given, 4 (1.6%) students indicated that instruction should be of higher quality than in 2019/20 and 14 (5.8%) students gave a response which was categorized as *Other*. Many respondents acknowledged that for some of their peers attending campus during COVID-19 crisis may not be possible; for example, 10 students who wanted in-person lectures asked that online resources be provided also in case students could not attend. Other students who expressed a preference for in-person teaching understood that it may not be possible and listed various online resources that could be used instead. The students who asked for a blended approach suggested that lectures could be given online supplemented with small-group tutorials on campus. Some of these students explained that they wanted live tutorials because of the possibilities for interaction and because of motivation:

Student 120: Compulsory Tutorials, maybe in person. Maths requires full attention, and it is hard to motivate yourself behind a screen.

Three students described a general *blended* approach to learning where they would be on campus for some days of the week and remain at home on the others. One of these students also offered suggestions about how the timetable could be restructured to cater for this blended approach:

Student 48: Online lectures with notes. But have tutorials on campus same day as labs.

Fourteen of the students who expressed a preference for online instruction said that they wanted lectures to be given live (through Zoom or Teams) and 45 asked for recorded lectures or short videos. Choosing between *live* or *recorded* online lectures is an interesting dilemma with echoes of pros and cons in the answers to many of the other survey questions. Students indicated that *live* lectures offer the chance to ask questions of the lecturer in real time, whereas a question that arises during a *recorded* lecture or video must be emailed to the lecturer.

Student 185: I personally found live lectures in other subjects more useful as students could ask questions and it would be explained to everyone rather than emailing a lecturer and having them explain it to only one student in a reply.

On the other hand, *recorded* lectures do not require real-time attendance and can be viewed multiple times:

Student 49: I can review all the videos and notes easily as they’re up permanently. This makes revising much easier. Also, if I’m not available at a certain time I don’t miss the lecture, I just watch the video when I’m free.

However, it was evident from the responses that whatever the format students value contact with lecturers and tutors:

Student 280: I think contact with the lecturer is very important and for them to either have pre-recorded videos or live lessons where they are teaching the topic. I found it very difficult to try and understand completely new topics by just reading notes—without anyone explaining them.

A hybrid system is also possible and was mentioned by six students, whereby lectures happen live (either in person or online) and are subsequently uploaded. Unsurprisingly, students’ responses to this question were heavily influenced by their experiences during the closures, with many students referring directly to facets of the delivery of their teaching in their answers.

A significant number of responses called for additional resources to be shared with students: *lecture notes* (*n* = 25), *short video clips* (*n* = 22), *exam questions* (*n* = 12), *worked examples* (*n* = 12) and *practice questions* (*n* = 4). Many of these resources are typically available for students and are popular tools for revision. Our analysis of responses to this question suggests that, this year, changes in terminal assessments caused students to seek sample papers and exam-style questions; changes to support services and tutorials resulted in a shortage of worked examples for students to study; and practice quizzes were used as elements of continuous assessments and were popular among students.

In a similar vein, the survey asked an open-ended question (Q11) about students’ preferences for methods of assessment in subsequent modules. Students’ preferences for assessment present a clear preference for *online terminal examinations* (*n* = 90) over *in person* (*n* = 9) (with continuous assessment) and reflect a growing appetite for *increased* continuous assessment (*n* = 30) and even continuous assessment *only* (*n* = 54) assessments. Within *online terminal examinations* are distinctions between *uploading solutions* (*n* = 45) and *multiple choice question* (*n* = 44) formats. Here, students appear to prefer the simplicity of the multiple choice question format, though they value ‘attempt’ marks that are only possible when lectures correct their entire exam script. The remaining labels were *open book* (*n* = 13), *improved standards* (*n* = 7) and *concerns about technology* (*n* = 7).

### 3.5. Limitations of the study

There are aspects of the study that should be taken into account when considering the results. We would like to remind the reader that the results were gathered and analysed in June 2020 and, as such, only pertained to the second semester of the 2019–2020 academic year. Though fully online learning continued, the approaches to teaching and assessment of instructors may have subsequently evolved to incorporate much of what is discussed in this research. As mentioned previously, advertising a survey during summer was not an appropriate way to recruit students studying at Institutes of Technology. Similarly, the timing may have elicited a greater proportion of responses from university students who are preparing for repeat examinations or students who may check their emails more regularly than others. This may impact how representative our results are. We acknowledge the shortcomings in the timing of the survey, offering only the time sensitive nature of the research as a mitigating circumstance. We acknowledge that we do not have information about how often the teaching methods in Table 2 were used in individual modules and therefore it is difficult to make inferences based on this data. Another aspect to be discussed is the wording of questions: we decided to omit the results relating to Q13, for example, due to possible ambiguities in the wording of the question.

## 4 Discussion

The results of our analysis of the data on the nature of teaching, studying and assessment during the closures in Ireland confirmed the widespread use of VLEs such as Moodle and Blackboard to provide access to a variety of resources to students. Typically, these resources consisted of recorded lectures or short videos, lecture notes, practice questions and worked examples. Live online lectures were also given to 47% of students who responded to the survey. Our data suggest that lecturers created a huge amount of online content in a very short time period. Assuming that much of this happened on an individual basis, we would like to highlight the opportunity for collaboration within and across tertiary level. Collating these resources for shared access could potentially reduce the amount of time lecturers spend developing materials, time that could be redirected towards other aspects of teaching and assessment as advocated by Crawford *et al.* (2020).

The low number of students who reported their lecturer directing them to pre-existing online resources may indicate that lecturers are unaware of such sites. Remedying this could also allow more time to be devoted to teaching and assessment. Some students seemed to struggle with asynchronous delivery, and we are reminded of Trenholm & Peschke (2020) who noted that fully online learning places a larger responsibility on students to read or view resources themselves. The asynchronous nature of recorded lectures and lecture notes uploaded to VLEs is also indicative of ‘domestication of new media’ (Engelbrecht *et al.*, 2020).

Tutorials were interrupted to a greater extent than lectures and support services even more so. In general, students seemed to prefer live tutorials to online discussion forums. One of the reasons for this was the immediacy of response, though the transient nature of live sessions may also be a factor: posting questions to an online forum attaches the student’s name to the query and oftentimes the question itself remains visible on the forum indefinitely. Furthermore, mathematics notation can often act as a barrier to communication in online settings (Trenholm & Peschke, 2020). We see evidence of this in student responses to the challenges they faced in Spring 2020 (Table 6) and in their reluctance to ask questions in discussion forums.

Students reported an increased homogeneity in the delivery of lectures, tutorials and support services. In the past, lectures, tutorials and support services have offered different atmospheres and methods of interaction that students engage with at various levels depending on what is effective for them. During the COVID-19 interrupted semester, however, all of these services migrated online and consisted of largely similar modes of delivery. This manifested itself in less satisfaction on the students’ end which centred on the loss of peer study and one-to-one support that occurs exclusively outside of lectures (typically in Mathematics Support Centres). This finding is in line with Croxton’s (2014), who found that student-instructor interactions are important both for student satisfaction and persistence.

We would like to highlight the importance of this in the context of mathematics education currently and suggest that tutorials and support services may be priorities in future blended approaches to learning that involve a gradual or staggered return to campus. This point is also evident in students’ opinions on what should happen in future semesters—many of them asked for live (either in person or via Teams/Zoom) lectures and tutorials and stressed that the opportunities for interaction with teaching staff and other students was of major importance to them. This was echoed by Meehan & Howard (2020) who reported that the sudden shift online adversely affected the more ‘interactive’ lectures (as perceived by students).

Our data showed a combination of continuous assessment and online terminal exams as the most frequently used method of assessment (either multiple choice question style or written with upload of solutions). Feedback on assessment would have been a welcome addition for many students whose interaction with lecturers and tutors reduced drastically. The technical difficulties that occurred during online assessments reported by students are particularly concerning for high stakes assessments. The problems involving poor connectivity and/or hardware (laptop/scanner) faults may be evidence of inequality affecting outcomes for students in a manner that is unique to the online setting. Responses to parts of Q12 (Table 1) showed a majority of students had access to the requisite hardware and internet connectivity to engage with lectures remotely, although many students were relying on their smartphones or tablets in order to access resources and to act as scanners. We note, however, that students without access to laptops and broadband are less likely to have responded to our survey, making our reported figures something of a ‘best case scenario’. It is concerning that nearly a quarter of the respondents did not have a quiet place to study or indeed to take their final examinations. Some improvements have been made recently in this area, with universities making quiet rooms available on campus so that certain students have a suitable location to take their examinations (even during subsequent university closures). In addition, the Irish government launched a scheme to help students to buy laptops. The access to reliable and fast broadband is a more difficult issue to solve in the short term, but has been flagged previously (Becker *et al.*, 2017).

The survey responses highlighted the increased isolation and lack of motivation brought on by the tertiary level closures. Unger & Meiran (2020) have reported this with respect to COVID-19 specifically and have called for students’ mental health to be monitored during epidemics. They also found that students lamented the reduction in contact with their lecturer, as did the students in our study, and others in the Irish context (Hill & Fitzgerald, 2020). Huang & Zhao (2020) state that people under the age of 35 are more likely to experience increased anxiety during the pandemic and, given the reduction in access to general support services such as counselling, we advise educators to be extra vigilant of students’ well-being. The students in our survey recommended that the feelings of isolation could be lessened if courses involved more interaction with instructors and with peers which is in agreement with the recommendations of Croxton (2014).

The importance of interactivity and communication to respondents is in keeping with more general characteristics of Gen Z students described by Dineva *et al.* (2019) and Engelbrecht *et al.* (2020), mentioned in the introduction. It seems that students’ ability to collaborate was impacted by the disruptions to peer working spaces reported in this work. Specifically, 57% of respondents to this survey reported that it was more difficult to communicate with peers; 66% said it was more difficult to engage in peer study; and 38% reported that the closures impacted their ability to study with peers ‘very negatively’. It is interesting to note that although it is likely that many of these students are part of the generation that uses social media the most, they still value the face-to-face nature of communication in lectures, tutorials and support centres.

Overall, the students show an understanding for the need for sudden and significant changes to have happened. They recognize the scale of the task this presented to lecturers and have provided suggestions for the improvement of teaching and learning in the future. When asked about returning to campus in the 2020/21 academic year, students showed a slight preference for distance learning over in-person classes, with various blended approaches mentioned by a minority of students. Finally, two particularly interesting comments that concern delivery of teaching upon partial return to campus are *More/All resources should be uploaded even when in-person delivery returns* and *Tutorials and support services should return to in person before lectures.* One approach to blended learning could be to facilitate some teaching in person while continuing to offer the same product online. This approach has been used by some American universities for 10 years (Bell & Federman, 2013) and would be an improvement on the quality of blended learning reported in this study. Prioritizing support services above lectures is supported by much of the data previously reported. With respect to future assessment, the majority of students still expressed a preference for final examinations, but some saw the value of increased continuous assessment in the future.

A concern in the open response questions that ended the survey (Tables 6–8) is about the clarification of expectations and standards for teaching and assessment. The labels *Clarify standards across departments/institution* and *Assessments need to be improved and standardized* capture the frustration of students with respect to how their modules were delivered and assessed post-closure. The comments refer to different experiences from module to module within and across departments. It is unrealistic to expect delivery and assessment to have been completely standardized for Semester 2 2019–2020, but perhaps conversations that clarify what is expected of lecturers, tutors and students should be facilitated where possible to ensure the highest quality of teaching and learning occurs moving forward. Trenholm & Peschke (2020) discuss the difference in assessment between fully online and face-to-face teaching in terms of expectations and administration. Our data report several issues with the running of online terminal examinations which were to be expected but have been described by Prince *et al.* (2009) over a decade ago when they noted the requirement for ‘additional human or computer resources requiring additional time and effort’ in such situations.

The move to online learning had some benefits for students; for example, for some, it removed the burden of commuting. However, the challenges for students with caring responsibilities and/or frontline jobs, students without access to the appropriate technology or infrastructures and students without access to an appropriate study space were difficult to surmount. Each of these circumstances present unique difficulties, and the issue of equal access in distance learning must be addressed. It is crucial that insights from students are heard and incorporated into the delivery and assessment of mathematics moving forward. Distance learning offers obvious cost-saving measures for institutions (building costs, scope to increase enrolment) and students (rent/commute), although there are also additional costs incurred by both groups. For example, implicit in online learning is access to the necessary equipment in an appropriate environment (discussed previously).

Hodges *et al.*’s (2020) description of emergency remote teaching encapsulates the initial COVID-19-related closures well, but we wish to highlight their inference that such an approach is only intended to be short term. At the time of writing, Irish universities are beginning their third semester of teaching in this manner and there is no doubt that the community has become more accustomed to the changes. With the rollout of vaccines ongoing, an end to COVID-19 related closures is in sight but the lessons we have learned as a community should not be discarded (Hodges *et al.*, 2020). Future events (e.g. weather and public health) will require emergency remote teaching to be used again.

This research was undertaken to give a voice to tertiary level students who were studying mathematics at the time of the COVID-19 related closures. We view students as equal stakeholders in the teaching and learning of mathematics at tertiary level and wanted to ensure their voices were heard as we move forward in a constant state of flux. The maturity, patience and insight offered were admirable. Staff and students were faced with many difficulties in March 2020 and our analysis has highlighted the areas of concern for students. In particular, our data have revealed the importance of personal contact with instructors and peers to undergraduates.

## Footnotes

1 It is worth noting that student responses to the survey were gathered during July and August 2020, and some of the data may have to be placed in the context of what was known and not known about how learning would occur at that time.

2 Though we use the term ‘tertiary level’ to describe higher education institutions in the text, ‘third-level’ is commonly used in Ireland. The survey questions used ‘third-level’ and so this language is retained in this paper wherever a question is stated verbatim.

## Notes

**Diarmaid Hyland** has a BSc in Science Education and a PhD in Mathematics Education. He is an Irish Research Council Postdoctoral Fellowship awardee based in the Department of Mathematics and Statistics at Maynooth University. His research interests are in the areas of undergraduate mathematics education and STEM teacher education.

## Notes

**Ann O'Shea** has a PhD in Mathematics and is a professor in the Department of Mathematics and Statistics at Maynooth University. She conducts research in undergraduate mathematics education.

## A. Appendix A. The Impact of COVID-19 on Students’ Learning

Institution:

Degree:

Year of study (i.e. 1^st^, 2^nd^, etc…):

Teaching and Learning

1. Which of the following has your lecturer used to deliver teaching during the COVID-19 closures:

a. Live, lectures hosted on an online client Yes **☐** No **☐**
b. Recorded lectures uploaded to a shared space Yes **☐** No **☐**
c. Written notes uploaded to a shared space Yes **☐** No **☐**
d. PowerPoint or similar presentations uploaded to a shared space Yes **☐** No **☐**
e. Worked examples uploaded to a shared space Yes **☐** No **☐**
f. Assignment/past exam question solutions uploaded to a shared space Yes **☐** No **☐**
g. Short videos addressing specific questions or procedures uploaded to a shared space. Yes **☐** No **☐**
h. Practice quizzes Yes **☐** No **☐**
i. Directed you to various online resources that existed pre COVID-19 Yes **☐** No **☐**

If none of the above, how was the module taught?

2. Were tutorials offered during the COVID-19 closures? If so, how? If not, what kind of support was available?

3. How have the COVID-19 third-level closures impacted your capacity to:

a. Learn mathematics generally
b. Learn through lecture
c. Learn through tutorial
d. Learn through maths support services
e. Independently study
f. Peer study
g. Communicate with your lecturer
h. Communicate with your peers
i. Communicate with student support services
j. Complete assignments and other forms of assessment

Very negatively **☐** Somewhat negatively **☐** No impact **☐** Somewhat positively **☐** Very positively **☐** Not applicable **☐**

4. How have the COVID-19 third-level closures impacted how often you use the following resources to support your learning:

a. Lectures
b. Lecture notes provided by the lecturer
c. other resources provided by the lecturer (for example, worked examples and short videos)
d. Maths support services
e. Prior exam questions
f. Practice quizzes
g. Online resources (from outside your institution)
h. Books

Very negatively **☐** Somewhat negatively **☐** No impact **☐** Somewhat positively **☐** Very positively **☐** Not applicable **☐**

5. How would you like your maths modules to be delivered next year? Refer to elements of Q1–4 if you wish.

Assessment

6. Which of the following did your lecturer use to assess learning during the COVID-19 third-level closures:

a. Continuous assessment **☐**
b. Online exam with multiple choice questions **☐**
c. Online exam with upload of solutions **☐**
d. No assessment was carried out post-closure **☐**

7. Did you feel that the assessment was fair, given the circumstances? Yes **☐** No **☐**

8. Did you receive feedback on any submitted work? Yes **☐** No **☐**

9. Were there any technical difficulties with any of the assessments? Yes **☐** No **☐**

If so, what happened and how was it addressed?

10. How did you prepare for your assessment during the COVID-19 closure?

Was your preparation different to usual? If so, how?

11. How would you like to be assessed for future modules if the restrictions remain in place? Refer to Q6–9 if you wish.

Personal experiences

12. Please indicate how you feel about the following statements

a. Where the appropriate precautions are taken, I feel comfortable attending lectures on campus next year.
b. Where the appropriate precautions are taken, I feel comfortable attending tutorials on campus next year.
c. Where the appropriate precautions are taken, I feel comfortable attending maths support services on campus next year.
d. It was easier to motivate myself to learn during the lockdown than before.
e. I felt more anxious about my learning during lockdown than before.
f. I felt isolated from my lecturer/class group. If so, how could this be avoided?
g. Did you have access to a quiet place to study?
h. Did you have access to a table/desk?
i. Did you have access to a PC or laptop?
j. Did you have access to a printer/scanner?
k. Did you have access to fast and reliable broadband?
l. Did you experience any technical problems? If so, what were they?

Strongly agree **☐** Somewhat agree **☐** Neutral **☐** Somewhat disagree **☐** Strongly disagree **☐** Prefer not to say **☐**

13. Please indicate how the changes in teaching and learning during the COVID-19 third-level closures changed your desire to study mathematics?

a. I feel much more inclined to study mathematics **☐**
b. I feel somewhat more inclined to study mathematics **☐**
c. I feel the same way about studying mathematics **☐**
d. I feel less inclined to study mathematics **☐**
e. I will avoid studying mathematics again where possible **☐**

14. In your opinion, what have been the challenging aspects of learning, teaching, and assessment in mathematics during the COVID-19 third-level closures?

15. In your opinion, what have been the positive aspects of learning, teaching, and assessment in mathematics during the COVID-19 third-level closures?

16. Please share any remaining comments and opinions you may have relating to the COVID-19 third-level closures and their impact on your learning of mathematics.
